# Survey of Zoonotic and Non-zoonotic Vector-Borne Pathogens in Military Horses in Lisbon, Portugal

**DOI:** 10.3389/fvets.2020.591943

**Published:** 2020-10-15

**Authors:** Hans-Peter Fuehrer, Ana Margarida Alho, Feodora Natalie Kayikci, Bita Shahi Barogh, Hugo Rosa, José Tomás, Hugo Rocha, Josef Harl, Luís Madeira de Carvalho

**Affiliations:** ^1^Department of Pathobiology, Institute of Parasitology, University of Veterinary Medicine, Vienna, Austria; ^2^CIISA - Centro de Investigação Interdisciplinar em Sanidade Animal, Faculdade de Medicina Veterinária, Universidade de Lisbon, Lisbon, Portugal; ^3^Guarda Nacional Republicana, Lisbon, Portugal; ^4^Department of Pathobiology, Institute of Pathology, University of Veterinary Medicine, Vienna, Austria

**Keywords:** equine piroplasmosis, military horses, *Theileria equi*, vector-borne diseases, zoonosis, Portugal

## Abstract

Vector-borne diseases of zoonotic and/or veterinary relevance have been increasingly reported in horses globally, although data regarding working and military horses is lacking. Portuguese military horses may constitute a risk group for these pathogens, as they frequently work outdoors in various regions of the country. This study included 101 apparently healthy horses belonging to the Portuguese National Republican Guard. Blood samples were analyzed to determine the presence and prevalence of piroplasms, *Anaplasmataceae, Rickettsia* spp., and filarioid helminths. Overall 32.7% of the horses gave positive results for *Theileria equi*. Two genotypes of *T. equi* were verified. No positive results were recorded for *Anaplasma* spp., *Rickettsia* spp., filarioid helminthes, and *Babesia caballi*. As equine piroplasmosis is a severe infectious tick-borne disease responsible for significant losses in equine production and with numerous impacts in the international movement of horses, adequate treatment, and preventive measures are needed to reduce exposure to vectors and future infections.

## Introduction

Vector-borne diseases (VBDs) have been increasingly reported in horses worldwide (1). Several equine VBDs are of zoonotic relevance and horses potentially serve as sentinels for human infections. The distribution and spread of vector-borne pathogens are limited by the presence of competent arthropod vectors (e.g., ticks, fleas, and mosquitoes) capable of transmitting these pathogens. Military horses may constitute a risk group for VBDs, as they frequently work outdoors in different areas and thus are exposed to vectors present there.

Equine piroplasmosis is a tick-borne disease of equids such as horses, donkeys, mules, and zebras, and is caused by the protozoan parasites *Theileria equi* and *Babesia caballi* (2). Infected animals may carry *B. caballi* for several years and *T. equi* for a whole lifetime (2).

*Theileria equi* (Laveran, 1901) Mehlhorn, Schein 1998 is one of the most important pathogens of horses in many parts of the world, including Southern Europe. Clinical signs range from asymptomatic to acute, subacute, and chronic cases with fever, anemia, inappetence, and spleno- and hepatomegaly. In severe cases, the infection can lead to death. Transplacental transmission from mares to fetuses can lead to abortion (3), causing significant losses in the equine industry. Additionally, international horse trade facilitates the spread to non-endemic areas (4). Currently, more than 20 potential tick vectors of *T. equi* are known (5, 6). Species of the genera *Dermacentor, Rhipicephalus*, and *Hyalomma* are known as competent vectors and several *Ixodes, Haemaphysalis*, and *Amblyomma* species are discussed to transmit the parasite as well (2). Various tick species that may act as vectors for equine piroplasms are present in mainland Portugal, namely *Rhipicephalus sanguineus, Rhipicephalus annulatus, Rhipicephalus bursa, Dermacentor marginatus, Hyalomma lusitanicum*, and *Hyalomma marginatum* (7, 8). Several genotypes of *T. equi* have been reported in different populations of equids (2, 9). There is evidence that specific genotype assortment occurs within the tick vectors (6). In Portugal, previous studies show different prevalence rates for *T. equi*, which may be associated with the kind of horses/facility involved, the study area and the diagnostic tests used. In horse stud farms in the Ribatejo region, central Portugal, using Complement Fixation (CF), seroprevalence was 45.3%, while using blood smears, the infection was prevalent on 42,6% of the examined horses, with a potential transuterine transmission in 80% of positive mares (10, 11). Further South, in Alentejo region, using CF and IFAT the seroprevalence for *Theileria equi* was 85.1% and recently, using qPCR/nPCR, 56% of the horses examined from the Lisbon and Alentejo areas showed a prevalence of 56% (12, 13).

Members of the family *Anaplasmataceae* (e.g., *Anaplasma, Neoehrlichia*, and *Ehrlichia*) are gram-negative, intracellular bacteria infecting domestic and wild animals, but also humans. *Anaplasma phagocytophilum* has been documented in various animals including horses and is the causative agent of equine, canine, and human granulocytic anaplasmosis. Various strains are known and horses can harbor strains of zoonotic potential (14). In Europe, the main vector of this tick-borne pathogen is *Ixodes ricinus*, which is also present in mainland Portugal (7). *Anaplasma phagocytophilum* has previously been detected in *I. ricinus* on Madeira and in *Ixodes ventalloi* in mainland Portugal (Baixa de Palmela, Setubal district) (15, 16). *Anaplasma phagocytophilum* is regularly reported in serology-based studies on horses in Europe including Portugal [e.g., (8, 16, 17)].

*Rickettsia* species are obligate intracellular, gram-negative bacteria transmitted by various types of arthropods [e.g., ixodid ticks are the main vectors of spotted fever group rickettsiae (18)]. Several studies have documented *Rickettsia* spp. in horses.

Filarioid nematodes are also parasites of horses in Europe. Because of their asymptomatic to minor symptomatic effects, these parasites are neglected and understudied. Adults of *Setaria equina* are located in the peritoneal cavities of horses. Microfilariae of this worldwide distributed helminth can be found in the peripheral blood (19). Mosquitoes of the genera *Culex* and *Aedes* are the vectors of *S. equina*. Another filarioid species parasitizing medial layers or outside layers of tissues within the artery wall of horses is *Onchocerca boehmi* (syn. *Elaeophora boehmi*) (20, 21).

Little is known about the risk of military horses regarding VBDs. In fact, few studies have been conducted so far and no surveillance mechanisms are in place to assess geographical range and prevalence in the country. Considering the emergence of VBDs in Europe, as well as the lack of data on this topic, an epidemiological study was conducted, in order to identify the presence and prevalence of the most significant bacterial and parasitic VBDs of zoonotic and/or non-zoonotic relevance in Portuguese military horses using molecular analysis followed by sequence analysis of positive DNA products.

## Materials and Methods

Overall 101 military horses (Puro Sangue Lusitano breed) belonging to the Portuguese National Republican Guard (GNR), stabled at guard facilities in metropolitan Lisbon were included in this study. Horses from various job sites were included (55 GNR Lisbon, 46 GNR Lisbon Braço de Prata Expo, 2 GNR Évora, 1 GNR Faro, and 1 GNR Tomar), and information on sex and age were determined. All horses were apparently healthy with no clinical signs compatible with VBDs. Horses were dewormed with moxidectin and praziquantel administered annually (with the exception of sportive horses, which were dewormed twice a year). Horses were not treated against tick infestation. Blood samples were collected at the jugular vein from apparently healthy horses in June (*n* = 55) and July (*n* = 46) in 2017.

A total of 50 μl of blood was spotted on Whatman® filter paper (VWR International GmBH, Vienna, Austria) and dried at room temperature. Afterward, each filter paper was sealed in an envelope separately. Filter papers were shipped to the University of Veterinary Medicine Vienna for molecular analysis. Sections of ~4 mm in diameter were cut out of the center of the blood spots with sterile blades. DNA was extracted with a modified chelex-based technique using InstaGene™ matrix (Bio-Rad Laboratories, Hercules, California) as established previously (22). Extracted DNA was stored at −20°C until further analysis. Samples were screened for the presence of DNA of various vector-borne pathogens of zoonotic and non-zoonotic relevance using specific broad-range PCR assays, under conditions reported previously [(23); Table 1] as follows: piroplasms (incl. *Babesia* and *Theileria*) within the *18S* rRNA gene [BTH-1F/BTH-1R; (24)]; *Anaplasmataceae* (incl. *Anaplasma, Ehrlichia*, and *Neoehrlichia*) within the *16S* gene [EHR16SD/EHR16SR; (26)]; *Rickettsia* spp. within the *23S*/*5S* rRNA gene [ITS-F/ITS-R; (27)] and filiarioid helminths targeting a fragment of the mitochondrial *cytochrome c oxidase subunit I gene* [*COI*; H14FilaCOIFw/H14FilaCOIRv; (28)]. Additionally, all samples were further analyzed with a *Babesia* specific PCR analysis [Babfor/Babrev; (25)]. PCR products were analyzed by gel electrophoresis on 2% agarose gels stained with Midori Green Advance DNA stain (Nippon Genetics Europe, Düren, Germany). Positive reaction products were commercially purified and sequenced at LGC Genomics GmbH (Berlin, Germany).

**Table 1:** Primer sequences and PCR protocols used for molecular analysis of vector-borne pathogens in military horses.

**Organism**	**Gene/Locus**	**Primer sequences (5^**′**^ → 3^**′**^)**	**Amplification protocol**	**Product size (bp)**	**References**
Piroplasms (*Babesia* spp., *Theileria* spp.)	*18S* rRNA	BTH-1F: CCTGAGAAACGGCTACCACATCT BTH-1R: TTGCGACCATACTCCCCCCA	94°C: 2 min, 40 cycles - 95°C: 30 s, 68°C: 1 min, 72°C: 1 min, 72°C: 10 min	~700	(24)
*Babesia* spp.	*18S* rRNA	B-For: GACTAGGGATTGGAGGTC B-Rev: GAATAATTCACCGGATCACTC	94°C: 2 min, 30 cycles - 95°C: 1 min, 53°C: 1 min, 72°C: 1 min, 72°C: 7 min	~650	(25)
Anaplasmataceae	*16S* rRNA	EHR16SD: GGTACCYACAGAAGAAGTCC EHR16SR: TAGCACTCATCGTTTACAGC	95°C: 2 min, 35 cycles - 94°C: 1 min, 54°C: 30 s, 72°C: 30 s, 72°C: 5 min	345	(26)
*Rickettsia* spp.	*23S/5S* rRNA	ITS-F: GATAGGTCGGGTGTGGAAG ITS-R: TCGGGATGGGATCGTGTG	96°C: 4 min, 35 cycles - 94°C: 1 min, 52°C: 1 min, 72°C: 2 min, 72°C: 3 min	350–550	(27)
Filarioid nematodes	mt *COI*	H14FilaCO1Fw: GCCTATTTTGATTGGTGGTTTTGG H14FilaCO1Rv: AGCAATAATCATAGTAGCAGCACTAA	95°C: 2 min, 30–35 cycles - 95°C: 1 min, 53°C: 1 min, 72°C: 1 min, 72°C: 5 min	724	(28)

### Phylogenetic Analysis of the *T. equi 18S* Sequences

To show the diversity of *18S* lineages of *T. equi*, Bayesian inference (BI) and Maximum likelihood (ML) trees were calculated based on the sequences of the present study, and data was mined from NCBI GenBank. The GenBank sequences were retrieved by performing a BLAST search on a 530 bp *18S* section of *Theileria* spp. To identify the lineages of *T. equi*, the sequences were aligned and sorted with MAFFT v.7 applying the default settings (29). The sequences were visually inspected using BioEdit (30) and all sequences were removed which did not cover the entire length, contained ambiguity characters, and/or obvious sequencing errors, resulting in a total of 220 *T. equi* sequences. The sequences of the present study and NCBI GenBank were combined and a sequence of *Theileria ovis* (AY533144) was added for outgroup comparison. All sequences were re-aligned with MAFFT v.7 applying the option “G-INS-i” and then collapsed to haplotypes using DAMBE (31). The alignment contained 49 unique *T. equi* lineages and 535 sites, of which all 15 gap positions were removed before the phylogenetic analysis. The best-fit substitution model for the 520 bp alignment was inferred using the model search implemented in the W-IQ-TREE web server [http://iqtree.cibiv.univie.ac.at/; (32)], resulting in the model HKY+G. A ML consensus tree was calculated from 1000 bootstrap replicates using W-IQ-TREE. A BI tree was calculated with MrBayes v.3.2.7 (33). The Bayesian analysis was run for 10 million generations (2 runs each with 4 chains, one of which was heated) and every thousandth tree was sampled. The first 25% of trees were discarded as burn-in and a 50% majority-rule consensus tree was calculated from the remaining 7,500 trees.

## Results

**Figure 1:**
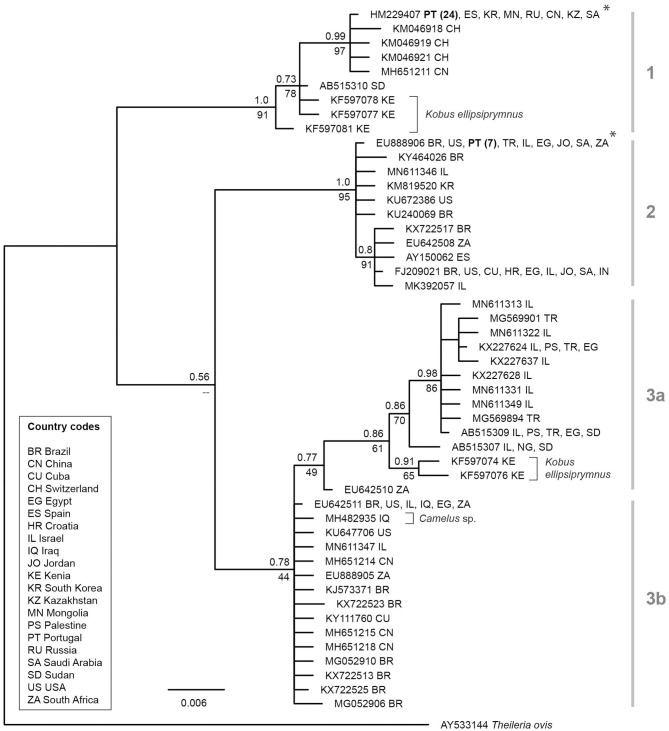
Bayesian inference tree of *T. equi* lineages based on 530 bp sections of the *18S*. The BI posterior probabilities and ML bootstrap values are indicated at most nodes. The scale bar indicates the expected mean number of substitutions per site according to the model of sequence evolution applied. Asterisks mark the lineages isolated from horses in Portugal in the present study. As outgroup a sequence of *Theileria ovis* (AY533144) was included.

In this study, 101 military horses were included of which 60 were males and 41 mares. The age ranged between 4 and 24 years (*x* = 12.4 years; x_med_ = 12 years). Overall 33 animals (32.7%; CI_95_: 24.3–42.3%) were infected with a vector-borne pathogen, namely *Theileria equi*. Seventeen males and 16 mares aged between 4 and 18 years (*x* = 10.5) gave positive signals at the molecular analysis for piroplasms. All of the positive horses came from Lisbon (23 at GNR Lisbon Braço de Prata Expo and 10 at GNR Lisbon). Two genotypes of *T. equi* were recorded of which 24 were genotype 1 and seven genotype 2. Both genotypes were present in GNR Lisbon Braço de Prata Expo and GNR Lisbon. The phylogenetic tree of the *18S* sequences shows three main sequence groups or clades, one of which may be divided into two sub-groups (Figure 1). The *T. equi* lineages identified in the present study clustered in the first and second clade. Of the 33 *T. equi* positive samples, 24 featured a lineage (genotype 1, 100% match with HM229407), which clustered in the first clade. This lineage and similar ones were isolated from horses in China, Kazakhstan, Russia, Mongolia, Saudi Arabia, South Korea, Spain, Sudan, and Switzerland. Three slightly distinct lineages (KF597077, KF597078, and KF597081) in this clade were isolated from the waterbuck *Kobus ellipsiprymnus* in Kenya. Seven horses sampled in the present study featured a *T. equi* lineage, which clustered in the second clade (Figure 1). This lineage (genotype 2, 100% match with EU888906) and similar ones were isolated from horses in Brazil, Croatia, India, Jordan, Egypt, Israel, Saudi Arabia, South Africa, Spain, Turkey, and the USA. The phylogenetic tree (Figure 1) features a third clade, which can be subdivided into two subclades (3a and 3b). Subclade 3a contains lineages isolated from horses in Egypt, Israel, Palestine, Turkey, Nigeria, and Sudan. All records come from the Middle East and Northern and Eastern Africa. Two slightly deviating lineages (KF597074 and KF597076) were isolated from the waterbuck *K. ellipsiprymnus* in Kenya. Subclade 3b features lineages isolated from horses in Brazil, China, Cuba, Egypt, Iraq, Israel, South Africa, and the USA.

Analyses for the presence of DNA of zoonotic agents (e.g., *Anaplasma* spp. and *Rickettsia* spp.) but also other non-zoonotic pathogens (e.g., *Babesia caballi* and filarioid helminths like *Setaria equina* and *Onchocerca boehmi*) gave negative results.

## Discussion

Prevalence of equine piroplasmosis vary in endemic regions in Europe for *T. equi* and *B. caballi*, depending on study design and diagnostic techniques used [IFAT, ELISA, PCR; (reviewed in 2)]. The prevalence of *T. equi* is generally higher than that for *B. caballi*. In healthy military horses included in this study, 32.7% (33/101) tested positive for the presence of DNA of *T. equi*. These data confirm a relatively high prevalence of *T. equi* in military horses in Portugal (mainly from the Lisbon area). In the Alentejo region (Southern Portugal) serological analyses with IFAT of 154 horses resulted in 85.1% positive for *T. equi* and 65.6% for *B. caballi* (12). Out of 73 “Puro Sangue Lusitano” horses from Vila Viçosa (Alentejo region, Southern Portugal) 53.4% were positive for *T. equi* using cELISA (34). Ribeiro et al. (8), combining serology and microscopy, reported *T. equi* in 19.1% and *B. caballi* in 11.7% of 162 horses in North of Portugal. Moreover, using PCR techniques, *T. equi* was detected in 56% of horses from central Southern Portugal that presented with clinical or subclinical signs (13). DNA of *T. equi* was found in two out of nine *Rhipicephalus bursa* male ticks collected from horses in the Comunidade Intermunicipal do-Ave (Northern Portugal), but because of the proof of DNA only, the vector capacity of this tick species remains unclear (35).

In the present study, horses between 4 and 18 years were infected with this parasite. It is known that the prevalence of equine piroplasmosis increases with age and that equids infected with *T. equi* remain life-long carriers of this parasite (2). Moreover, it is discussed that male and female horses have different susceptibilities to infection, but the results of various studies are contradictory. Host activity also increases the chance for horses to become infected (2). Grazing was documented to double the risk of becoming infected with *B. caballi* and *T. equi* (36). Outdoor or mixed indoor/outdoor type of housing was also reported as a risk factor for *T. equi* (8). Military horses included in this study are regularly outside, but are also stabled at military facilities. However, regular travel activity to various regions in the country increases the possibility of tick contact. Furthermore, the type of horse breed may also influence the risk of *T. equi* infections (37).

Interestingly we were not able to detect DNA of *B. caballi* in military horses from this study, although an additional *Babesia-*specific PCR was run. Previous studies have shown that horses seropositive for *B. caballi* (but also *T. equi*) are often not positive at PCR analysis (38). However, it is recommended to run cELISA, IFAT, and PCR to assure the identification of acute and chronic equine piroplasm infections (39). Recent research carried out on Lisbon horses, found a 7% prevalence for *Babesia* spp., meaning it may be a residual, but persistent agent, in this area (13).

*Anaplasmataceae* were not detected in the blood samples of healthy horses analyzed in this study. Using serology, *A. phagocytophilum* has previously been confirmed in nine of 302 horses from mainland Portugal (16) and 13% of 162 horses in Northern Portugal (8). However, it has been shown that seropositivity to *A. phagocytophilum* was significantly higher if compared to PCR results [e.g., (40)]. Therefore, we cannot exclude that horses in this study had previously been in contact with this pathogen. It is discussed that climate change might increase the threat of disease in European horse populations (41).

Although no horses were positive for *Rickettsia* spp. in this study, several *Rickettsia* species have been documented in horses in Europe. Spotted fever-group rickettsiae were reported in 15.03% of 479 horses in Italy using serology (17). Using IFAT, *Rickettsia helvetica* was documented in 36.5% of 63 horses in Sweden (42). In another study, *R. helvetica* and *R. monacensis* were confirmed in ticks collected from ponies in Poland using PCR techniques (43). On Corsica, the presence of *Rickettsia slovaca* and *Rickettsia aeschlimannii* in ticks collected on horses was confirmed (44).

DNA of microfilaria of filarioid helminths was not detected in the present study. However, although infections with these parasites are mainly asymptomatic and neglected, they have been reported in horses in several regions in Europe. In Hungary, microfilariae of *S. equina* were reported in 9.2% (18/195) of horses analyzed, and infection was associated with the presence of still waters nearby (45).

Although only one parasite species was documented, it can be concluded that *T. equi* is among the most important vector-borne transmitted agents infecting horses in Europe. As equine piroplasmosis is a severe infectious tick-borne disease responsible for significant losses in equine reproduction, and with numerous impacts on the international movement of horses, treatment and preventive measures are needed to reduce exposure to future infections.

## Data Availability Statement

The 18S sequences of *Theileria equi* were deposited in NCBI GenBank under the accession numbers MT767139-MT767169.

## Ethics Statement

This animal study was reviewed and approved by GNR–5373/17/CDF/GAB. Whenever possible, blood sampling was performed during normal sanitary surveys for the GNR horse population. Written informed consent was obtained from the owners for the participation of their animals in this study.

## Author Contributions

H-PF, AA, and LM contributed to the conception and design of the study. AA, HRos, JT, and HRoc conducted field and laboratory work. FK and BS conducted the lab work. H-PF and JH analyzed the sequences and performed the phylogenetic analysis. H-PF, AA, JH, and LM wrote and reviewed the manuscript. All authors read and approved the final manuscript.

## Conflict of Interest

The authors declare that the research was conducted in the absence of any commercial or financial relationships that could be construed as a potential conflict of interest. The reviewer CG declared a past co-authorship with one of the author H-PF to the handling editor.
